# Associations of childhood exposure to malaria with cognition and behavior outcomes: a systematic review protocol

**DOI:** 10.1186/s13643-020-01434-2

**Published:** 2020-08-09

**Authors:** Andrew Sentoogo Ssemata, Jacquelline Ann Nakitende, Simon Kizito, Elizabeth C. Whipple, Paul Bangirana, Noeline Nakasujja, Chandy C. John, Megan S. McHenry

**Affiliations:** 1 Department of Psychiatry, School of Medicine, College of Health Sciences, Makerere University, P. O. Box 7072, Kampala, Uganda; 2 Department of Mental Health and Community Psychology, School of Psychology, Makerere University, Kampala, Uganda; 3 Ruth Lilly Medical Library, School of Medicine, Indiana University, Indianapolis, Indiana, USA; 4 Department of Pediatrics, School of Medicine, Indiana University, Indianapolis, Indiana, USA

**Keywords:** Children, Malaria, Cognition, Behavior, Uncomplicated, Asymptomatic, Severe, Assessment

## Abstract

**Background:**

Malaria is one of the major contributing risk factors for poor development of children living in low- and middle-income countries (LMICs). However, little is known about the specific domains of cognition and behavior that are impacted by malaria, the extent of these deficits, and the different types of the malaria spectrum that are associated with these deficits. The objective of this systematic review is to determine the association of the different types of malaria infection on cognition and behavioral outcomes among children living in LMICs.

**Methods and analysis:**

We will systematically search online bibliographic databases including MEDLINE (via PubMed), CINAHL (via EBSCO), PsycINFO (via EBSCO), Embase, and The Cochrane Central Register of Controlled Trials (CENTRAL) as well as Google Scholar and bibliographies of pertinent articles. We will include studies with a comparison group (e.g., clinical trials, cohort, observational, cross-sectional case–control, and controlled before and after or interrupted–time–series studies) involving children under 18 years of age living in LMICs, as determined by the World Bank criteria, with either an active malaria infection or history of malaria. Included articles must also measure cognitive and/or behavior outcomes determined by standardized psychological assessments (questionnaire-based scales and or neurocognitive assessments). Studies will be excluded if they are not in English, lack a control group, take place in a high-income country, or if a standardized instrument was not used. Two reviewers will independently review all articles to determine if they meet eligibility criteria. Any conflicts will be resolved after discussion with a third reviewer. When a list of included articles is finalized, two reviewers will extract data to populate and then cross check within an electronic table. Risk of bias and the strength of evidence and recommendations will be assessed independently using the Grading of Recommendations Assessment, Development and Evaluation (GRADE) criteria, and a final score will be given upon consensus. For sufficiently homogeneous data on measured outcomes in multiple studies, we will investigate the possibility of pooling data to perform a meta-analysis.

**Discussion:**

This systematic review will evaluate the evidence of the association of malaria on the cognitive and behavioral outcomes. Findings from this planned review will generate insight on the domains affected by the different forms of malaria infection and may inform subsequent malaria interventions and future research in pediatric care.

**Systematic review registration:**

PROSPERO CRD42020154777

## Background

Malaria remains a public global health challenge that disproportionally affects children who already face multiple other risk factors for growth and development, such as poverty and malnutrition especially in low and middle-income countries (LMICs) [1, 2]. Between 300-600 million people suffer from malaria each year, and 90% of these cases occur in young children in sub-Saharan Africa [3]. Over 300,000 of children infected with malaria will die of the disease [4, 5]. Those who survive often suffer multiple morbidities with severe and complicated outcomes including increased risks of neurological, cognitive, and behavioral deficits [6–10]. With the high numbers of malaria infection worldwide, many children are at increased risk of cognitive and behavioral impairments. By not reaching their full developmental potential, these children are estimated to have a 20% deficit in their adult incomes, which affects families, communities, and countries [11].

A review by Holding and Snow [12] reported that the effect of malaria in childhood has subtle to profound effects on cognition and behavior linked to the negative brain injury consequences and the impact on performance exacerbated by chronic or recurrent malaria infection, anemia, and undernutrition [12]. A systematic review by Kihara, Carter [8] reported potential deficits in cognition: attention, memory, visuospatial skills, language, and executive functions after *Plasmodium falciparum* infection. The review suggests there is strong evidence that direct brain damage during malaria episodes causes neuro-cognitive impairment [8]. Additionally, a recent review by Fernando, Rodrigo [10] showed that few studies have assessed the impact of malaria on cognitive functions. The review found significantly impaired cognitive abilities and school performance among malaria groups before and after treatment of acute illness [10]. While these reviews brought attention to this critical issue, they were published over 10 years ago and lack differentiation regarding severity or different forms of malaria infection.

Furthermore, no reviews have evaluated the effects of *Plasmodium falciparum* on behavior, although persisting immediate and long-term behavioral problems have also been recorded in children surviving malaria [13–15]. A retrospective study conducted to assess the behavior problems in children aged 1-6½ years that had survived cerebral malaria with severe neurological sequelae who had suffered cerebral malaria at the ages of 5 months-4 years found them to have behavior problems that included inattention and impulsivity, aggression, ADHD with hyperactivity, destructibility, running away from home, autistic spectrum disorders, feeding difficulties and self-injurious behaviors [15]. Moreover, in a Ugandan study, children less than 5 years who had cerebral malaria and severe malaria anemia were found to higher externalizing and internalizing problems compared to the control children [13].

Important to note is that malaria is considered an independent causative factor for cognitive and behavioral impairment however the exact pathological mechanisms and the link between malaria and cognitive and behavior deficits remains unclearly defined [10]. One possible explanation is that parasitic sequestration within cerebral circulation causes biochemical changes in the central nervous system [10, 12]. This causes hypoxia, hypoglycemia, multiple, prolonged, generalized, or focal seizures; intracranial hypertension, severe metabolic derangement, and coma leading to damage of the frontal lobes, medial temporal lobes, and the hippocampal system [10]. Another possible explanation is that malaria induces acute neurological complications as a result of vascular obstruction, brain hyper-inflammation, reduced cerebral blood flow, and disruption of the blood-brain barrier (BBB) with consequent axonal damage and demyelination responsible for behavioral, and cognitive impairment [16]. These mechanisms have however been thought to be dependent on factors like the nature of parasite involved, the level of parasitization, infection, intensity, and the age of the host at infection [12]. It is important to note that these mechanisms may not cut across the different malaria types and thus the possibility of different outcomes for different malaria types.

Limited literature currently exists regarding what specific domains of cognition and behavior may be negatively impacted by malaria infection and the extent of that impact. Furthermore, it is unclear how other potential factors, such as varying intensities of malaria infection, may impact cognition and behavior. While national malaria control programs and malarial campaigns aim to reduce the number of malaria-associated morbidity and mortality [17, 18], many children surviving malaria in LMICs continue to present with cognitive and behavioral deficits. In recent years, cognitive and behavioral assessments are increasingly performed post-malaria infection, in both clinical and research settings. However, a succinct synthesis of these data has not been performed to describe the impact that various forms of malaria infection have on cognitive and behavioral outcomes in children, as well as other factors that may be contributing to cognitive and behavioral deficits in this population.

This systematic review is necessary to help find, appraise, and summarize the current evidence that malaria infection is associated with cognitive and behavioral performance and may provide evidence to inform clinical care, identify knowledge gaps, and highlight further areas for investigation. We focus on LMICs where malaria is still greatly endemic with moderate and high transmissions and malaria burden [1, 2]. For example in 2018, nineteen countries in sub-Saharan Africa and India carried almost 85% of the global malaria burden with six countries (Uganda Mozambique, Nigeria, the Democratic Republic of the Congo, Côte d’Ivoire, and Niger) bearing more than half of all malaria cases worldwide [2]. Additionally, quality of malaria care among children varies widely in endemic LMICs with fragile health systems creating major health and development challenges (1) as not all children with apparently the same brain insult have the same outcome. This is notwithstanding the fact that LMICs receive the highest percentage of international funding for malaria (85% for low income and 61% for lower-middle-income countries in 2018) (2) proposing the need for further exploration.

### Objectives

The objective of this systematic review is to examine the association of malaria infection on cognition and behavior outcomes among children living in low- and middle-income countries (LMICs). We will achieve this objective by addressing the following research questions:

- Which cognitive and behavioral domains are negatively impacted by malaria infection?
- To what extent are these domains impacted?
- Which forms of malaria are associated with specific deficits in cognition and behavior?

## Methods and analysis

We developed our methods following the instructions of the Preferred Reporting Items for Systematic Reviews and Meta-Analysis (PRISMA) guidelines for which the systematic review will be written. This systematic review was also written in accordance with the PRISMA for systematic review protocols (PRISMA-P) statement [19, 20] (see Additional file 1). This systematic review has been registered under the International Prospective Register of Systematic Reviews (PROSPERO; registration number: CRD42020154777).

### Eligibility criteria

Quantitative studies that have used standardized psychological tools to assess cognitive and behavioral outcomes among children surviving malaria will be included. Studies will be selected according to the following eligibility (inclusion/exclusion) criteria (see Additional file 2).

#### Inclusion and exclusion criteria

We will include published randomized controlled trials (RCTs), longitudinal studies, case-control studies, cohort studies, cross-sectional studies, observational studies, controlled before and after, or interrupted–time–series studies and case series including > 5 individuals. The inclusion of longitudinal studies will allow us to evaluate the potential impact of malaria on cognition and behavior over time. We will include only human studies of children below 18 years of any gender with malaria infection by laboratory diagnosis (i.e., microscopic diagnosis, molecular diagnosis, antigen detection, and serology). We will not exclude studies by community or hospital setting. However, it is likely that most studies will investigate patients undergoing treatment and therefore be in hospital settings. We will include studies with a comparator or a comparison group that report cognitive and/or behavior score as determined by a standardized psychological assessment as a primary outcome.

We will only include studies published in English or with an English translation as we do not have access to scientific translation services. The publication will be considered if the study was conducted in a LMICs in Asia, Africa, and Central and South America, as determined by the 2020 World Bank criteria [21]. The World Bank classifies countries into four income groupings (low, lower-middle, upper-middle, and high) basing on their income/economies. Income is measured using gross national income (GNI) per capita, in U.S. dollars converted from local currency for the previous calendar year [21, 22]. For the current criteria and list of eligible countries, see Additional file 2: Table 2.

We will exclude studies that (1) consider malaria infection determined by clinical diagnosis only (patient symptoms and on physical examination); (2) human studies with participants older than 18 years of age and do not assess cognitive and or behavioral function as an outcome; (3) animal studies; (4) studies without a control or comparison group; (5) studies in high-income countries or not within LMICs, as determined by the World Bank criteria. We will exclude non-English studies, reviews, opinion pieces, and letters to the editor, commentaries, abstracts, and case series including < 5 individuals.

We will use the participant/population, intervention, comparator, outcomes, timing, setting/study design (PICOTS) typology to describe the important key question and clarify the context relevant to our review as below.

##### Participants

The target population for inclusion is children (≤ 18 years) with active or recent malaria (within 12 months) infection. Inclusion of longitudinal studies will allow us to evaluate the potential impact of malaria on cognition and behavior over time [23].

##### Intervention/exposure/target condition

We will include any active malaria infection or any recent malaria infection that has occurred within 12 months of the cognitive and behavioral assessments. When possible, we will classify the malaria according to the following definitions:

Asymptomatic malaria is a form of *Plasmodium* malaria infection that lacks typical clinical symptoms but has sub-microscopic parasite densities detectable by microscopy, rapid diagnostic test (RDTs), or molecular methods [24]. Asymptomatic malaria is commonly a sub-microscopic infection (does not necessarily produce gametocytes chiefly detectable by molecular methods than microscopy).

Uncomplicated malaria is a form of *Plasmodium* malaria infections that are accompanied by fever and/or other symptoms like nausea, vomiting, muscle aches, abdominal pains, chills, and sweat that are indicative of malaria. These infections are, almost without exception, detectable by microscopy or rapid diagnostic test [24].

Severe malaria is almost exclusively caused by *Plasmodium falciparum* infection characterized by prostration, impaired consciousness, respiratory distress (acidotic breathing), multiple convulsions, circulatory collapse, pulmonary edema (radiological), abnormal bleeding, jaundice, hemoglobinuria, severe anemia with quick progression to life-threatening disease [25, 26]. However, *P. vivax* [24] and *P. knowlesi* [27, 28] can also cause severe disease.

If the specific form of malaria infection is not clear from the publication, it will be labeled as “general malaria infection.” If the study cohort contains individuals with different forms of malaria infection, it will be labeled as “mixed malaria infection.” The purpose of the classification is to enable reporting on how the different forms of malaria affect cognitive and behavioral outcomes and any differences/variations in outcomes observed.

##### Comparator

Studies with a comparator or control group used against the cognitive and behavioral assessment as one of the key outcomes.

##### Outcome

The primary outcome of this systematic review is an evaluation of cognitive and/or behavior outcomes as determined by a standardized psychological assessment (questionnaire-based scales and or neurocognitive assessments) as seen in Additional file 2: Table 3.

##### Timing

For this review, timing will reflect the length of time that separates the malarial illness and the completion of the outcome assessment. Additionally, this outcome may be assessed at a single time point or at multiple follow-ups, both of which will be considered.

##### Setting and study design

Only studies undertaken in low- and middle-income countries will be eligible for inclusion, due to the high burden and receptivity of malaria infection in these settings. Income classification of countries will be according to the World Bank criteria [21, 22]. We will include study designs like randomized controlled trials (RCTs), longitudinal studies, case-control studies, cohort studies, cross-sectional studies, observational studies, case series including > 5 individuals.

##### Language

Studies published in the English language and available in full text will be eligible for inclusion. Studies published in any language other than English will not be included. This criterion is due to the limited resources available in performing this review.

### Information sources

We will systematically search MEDLINE (via PubMed), Cumulative Index to Nursing and Allied Health—CINAHL (via EBSCO), PsycINFO (via EBSCO), Embase, and The Cochrane Central Register of Controlled Trials (CENTRAL). We will also search the reference lists of included studies to identify other studies. We consulted a medical librarian at Indiana University to develop our search strategy. Our final search terms include text words; malaria (asymptomatic, uncomplicated, and severe), cognitive, behavior, child development; MeSH terms for cognition, behavior, child, malaria with the child filter (birth-18 years) (see Supplement 1). We will also hand search the bibliographies of relevant studies, studies that have cited those included in our review and review articles, as well as search Google Scholar and relevant websites, i.e., WHO, Malaria consortium and OpenGrey (www.opengrey.eu/—for grey literature) for additional potentially eligible articles for inclusion. Our search will not be restricted by earlier publication date and length of follow-up in order to compare outcomes across time. We will include studies published until the time of the search is performed. Note: Our preliminary scoping of the literature suggested no relevant citations would be retrieved prior to 1920.

### Study screening, selection, and data extraction

Studies will be included only if they meet the full inclusion criteria above and not met any exclusion criteria. The abstracts and full-text articles retrieved using the search strategy will be imported into the Endnote software and duplicates will be removed. Using a two-tier approach, two of the authors will independently screen studies for inclusion at two levels: first at title/abstract level and then at full-text based on the eligibility criteria. We will then screen full-text articles of studies that will have met eligibility at title and abstract screening. The two reviewers will be blinded to each other’s screening results during this screening process. For any discrepancies between the two reviewers at both title/abstract level and then at full-text level, another author will serve as a third rater to create a consensus. The two reviewers will each independently extract data from one-half of all the full-text articles of the included studies and populate the tables using an Excel screening and data extraction spreadsheet developed a priori to capture study details (e.g., authors, year, country, setting, length of follow-up); sample characteristics (e.g., age, gender, number of participants included), study design (e.g., randomized controlled trials (RCTs), case control, cohort, cross sectional), descriptions of how malaria, cognition, and behavior are assessed (tests and cut-off values used), and study outcomes (see Additional file 2: Table 4). We will then cross-check the other half of the data tables to guarantee accuracy. The Excel spreadsheet and Endnote library will be used to manage records and data throughout the review.

### Assessment of risk of bias and grading strength of evidence

To reduce the risk of bias, studies will be assessed independently by two reviewers according to inclusion and the exclusion criteria and in case of discrepancies, it will be resolved by consensus by a third reviewer. Technical experts (content experts) will be consulted when needed. We will assess for key aspects like allocation concealment, random sequence generation, and attrition for randomized trials; choice of controls for case–control studies; similarity of baseline characteristics, sample size, control group for observational study designs; sampling strategy, response rates for cross–sectional studies; attrition for cohort studies. We will conduct sensitivity analyses by risk of bias, using a fixed-effects model in order to explore the robustness of the results of our primary outcomes. To assess reporting bias, if the number of included studies is more than 6, we will use a funnel plot and Kendall’s test to explore publication bias. To evaluate the methodological quality and strength of evidence on the topic, we will use the Grading of Recommendations Assessment, Development and Evaluation (GRADE) approach [29, 30]. Two raters not involved in the screening process will independently assess the strength of evidence in five areas: study design, quality, consistency, directness, and precision. The raters will add or subtract points for each of these categories in line with GRADE guidelines. We will rate the overall quality of evidence for each outcome as “High,” “Moderate,” “Low,” or “Very Low.” By adding or subtracting points, GRADE helps us assess whether further evidence from newly published studies would change the conclusions of the review. The raters will resolve discrepancies through consensus and involvement of a third rater. We will develop a summary of findings, tables for each outcome, and identify areas for further research.

### Data synthesis and statistical analysis

We will narratively synthesize the extracted data from all included studies to provide a narrative account of the data extracted from the included studies. A PRISMA flow chart will be developed to show each level of the review process. First, we will use descriptive statistics and where possible Forest plots to summarize included studies and structured synthesis of data. Group-specific sample sizes, means, and SDs will be abstracted from included articles. Based on the fact that results of a single study can be either published in different or multiple publications, caution will be taken to ensure that the unit of analysis is the study rather than the different articles in order to avoid over or undercounting studies.

It is imperative to note that the use of a wide variety of often, not well-validated assessment tools are linked to the lack of culturally sensitive and appropriate tools to identify and assess cognitive and behavioral outcomes in children in LMICs [31]. This may create complexity in compatibility and comparison between studies in our systematic review. However, for 2 or more subsets of studies that are sufficiently homogeneous in terms of sample characteristics, same assessment measures of cognition and behavior, and methods (e.g., design, setting, length of follow-up), a meta-analysis will be considered [32]. The meta-analysis will be conducted using the Cochrane Collaboration Review Manager Software package (RevMan Version 5.3). We will consider heterogeneity (variation across studies) and consider a random-effects meta-analysis that assumes that the underlying effects follow a normal distribution. We will synthesize and transform effect size measures appropriately; for binary outcomes, we will use odds ratios and risk ratios and for continuous outcomes, we will use standard mean differences (Cohen’s d, Hedges’ g). We will explore methodological and statistical heterogeneity between studies with higher percentages signifying higher variation across studies using Cochran’s Q and quantify this using the *I*-squared statistics. In the absence of statistically significant heterogeneity, we will pool quantitative data using the random-effects model [33] and explore the robustness of the results of our primary outcome by conducting sensitivity analyses by risk of bias, using a fixed-effects model.

## Discussion and outcomes

The systematic review will provide meaningful insights on the association of malaria on cognitive and behavioral outcomes of children, including the adverse effects of post-malaria survival. Additionally, the review will inform strategies for the prevention and management of adverse outcomes associated with malaria among children in endemic LMICs. The findings may guide further research in addressing the current gaps in knowledge and limitations regarding cognitive and behavioral outcomes associated with malaria episodes.

This systematic review will be the first to examine a full range of both cognitive and behavioral outcomes in children in malaria-endemic and LMICs, as well as explore various forms of malaria infection may impact outcomes. Findings from this review may support clinicians, health experts, and policymakers develop guidelines to minimize deficits and impairment due to malaria infection; better inform policy; and develop interventions to improve outcomes for children surviving malaria.

### Ethics and dissemination

We will not seek for ethical approval as this is a systematic review protocol. We will synthesize literature on the cognitive and behavioral outcomes of children surviving malaria. The findings of this review will be shared electronically and in-print through conference presentations and peer-reviewed publications to provide information to scientists when developing guidelines for managing the outcomes and understanding the burden of malaria in LMICs.

## Supplementary information

**Additional file 1.** PRISMA-P 2015 Checklist

**Additional file 2:Table 1.** Inclusion and exclusion criteria. **Table 2.** Low and middle income countries based on the 2020 World Bank criteria (The World Bank. How does the World Bank classify countries New York: The World Bank; 2020 [cited 2020 30th April 2020]. Available from: https://datahelpdesk.worldbank.org/knowledgebase/articles/378834-how-does-the-world-bank-classify-countries). **Table 3.** Standardized tools and outcome measures for cognition and behaviour domains. Table 4: Data extraction/data charting tool

**Additional file 3.** Supplement

## Data Availability

Not applicable

## Abbreviations

BBB: Blood-brain barrier
CM: Cerebral malaria
GRADE: Grading of Recommendations Assessment, Development and Evaluation
GNI: Gross national income
LMICs: Low and middle-income countries
*P. falciparu*: *Plasmodium falciparum*
PICOTS: Participant/population, intervention, comparator, outcomes, timing, setting/study design
PRISMA: Preferred Reporting Items for Systematic Reviews and Meta-Analysis
PROSPERO: International Prospective Register of Systematic Reviews
RCTs: Randomized controlled trials
RDTs: Rapid diagnostic tests
SMA: Severe malaria anemia
