# Supplementary material for: Associations of childhood exposure to malaria with cognition and behavior outcomes: a systematic review protocol
Source: Syst Rev. 2020 Aug 9;9:174. doi: 10.1186/s13643-020-01434-2 (PMC7416398; doi:10.1186/s13643-020-01434-2)
Supplement: Supplementary file 2 — Additional file 2:Table 1. Inclusion and exclusion criteria. Table 2. Low and middle income countries based on the 2020 World Bank criteria (The World Bank. How does the World Bank classify countries New York: The World Bank; 2020 [cited 2020 30th April 2020]. Available from: https://datahelpdesk.worldbank.org/knowledgebase/articles/378834-how-does-the-world-bank-classify-countries). Table 3. Standardized tools and outcome measures for cognition and behaviour domains. Table 4: Data extraction/data charting tool [file 13643_2020_1434_MOESM2_ESM.docx]

**Table 1: Inclusion and exclusion criteria**

| **Inclusion criteria** | **Exclusion criteria** |
| --- | --- |
| - Studies that report on cognitive and behavioural outcomes | - Studies with no evidence on cognitive and behavioural outcomes |
| - Studies based on participants up to18 years | - Studies based on participants above18 years |
| - Studies reporting using standardized methods of measuring cognition and behaviour | - Studies reporting other non-standardised methods of measuring cognition and behaviour |
| - Be in English language | - Be in other languages |
| - Be available in full text | - Abstracts, newsletters, reviews |
| - Must have been published between 1920 to-date | - Studies published before 1920 |
| - All study design |  |

**Table 2: Low and middle income countries based on the 2020 World Bank criteria^[[1]](#footnote-1)^**

| **LOW-INCOME COUNTRIES (GNI per capita $1,025 OR LESS IN 2018)** | | | |
| --- | --- | --- | --- |
| Afghanistan | Ethiopia | Malawi | South Sudan |
| Benin | Gambia, The | Mali | Syrian Arab Republic |
| Burkina Faso | Guinea | Mozambique | Tajikistan |
| Burundi | Guinea-Bissau | Nepal | Tanzania |
| Central African Republic | Haiti | Niger | Togo |
| Chad | Korea, Dem. People's Rep. | Rwanda | Uganda |
| Congo, Dem. Rep | Liberia | Sierra Leone | Yemen, Rep. |
| Eritrea | Madagascar | Somalia |  |
|  |  |  |  |
| **LOWER-MIDDLE INCOME COUNTRIES (GNI per capita $1,026 TO $3,995 IN 2018)** | | | |
| Angola | El Salvador | Micronesia, Fed. Sts. | Solomon Islands |
| Bangladesh | Eswatini | Moldova | Sudan |
| Bhutan | Ghana | Mongolia | Timor-Leste |
| Bolivia | Honduras | Morocco | Tunisia |
| Cabo Verde | India | Myanmar | Ukraine |
| Cambodia | Indonesia | Nicaragua | Uzbekistan |
| Cameroon | Kenya | Nigeria | Vanuatu |
| Comoros | Kiribati | Pakistan | Vietnam |
| Congo, Rep. | Kyrgyz Republic | Papua New Guinea | West Bank and Gaza |
| Côte d'Ivoire | Lao PDR | Philippines | Zambia |
| Djibouti | Lesotho | São Tomé and Principe | Zimbabwe |
| Egypt, Arab Rep. | Mauritania | Senegal |  |
|  |  |  |  |
| **UPPER-MIDDLE-INCOME COUNTRIES (GNI per capita $3,996 TO $12,375 IN 2018)** | | | |
| Albania | Cuba | Kazakhstan | Romania |
| Algeria | Dominica | Kosovo | Russian Federation |
| American Samoa | Dominican Republic | Lebanon | Samoa |
| Argentina | Equatorial Guinea | Libya | Serbia |
| Armenia | Ecuador | Malaysia | Sri Lanka |
| Azerbaijan | Fiji | Maldives | South Africa |
| Belarus | Gabon | Marshall Islands | St. Lucia |
| Belize | Georgia | Mauritius | St. Vincent and the Grenadines |
| Bosnia and Herzegovina | Grenada | Mexico | Suriname |
| Botswana | Guatemala | Montenegro | Thailand |
| Brazil | Guyana | Namibia | Tonga |
| Bulgaria | Iran, Islamic Rep. | Nauru | Turkey |
| China | Iraq | North Macedonia | Turkmenistan |
| Colombia | Jamaica | Paraguay | Tuvalu |
| Costa Rica | Jordan | Peru | Venezuela, RB |

**Table 3: Standardized tools and outcome measures for cognition and behaviour domains**

| **Outcome variable** | **Assessment measure** |
| --- | --- |
| **Cognitive domain** | |
| Cognition, language, learning, planning, simultaneous processing, sequential processing, crystallised intelligence, spatial memory, visual abstraction, sequencing and reasoning, mental processing | Kauffman Assessment Battery for Children (KABC) |
| Communication, gross motor, fine  motor, problem solving, and personal-social development | Ages and stages questionnaire |
| Cognition, language, motor function | Bayley Scales of Infant development (1,2,3 and 4^th^ Editions) |
| Cognition, language, fluid intelligence, memory, intellectual ability, Attention, Organization Skills, Impulse Control Activity, visualisation | Leiter-R or Leiter International Performance Scale |
| Language development, comprehension, word production, gestures, grammar | MacArthur-Bates Communicative Development Inventories (CDI) |
| Cognitive and motor development, verbal memory, verbal fluency, Word Knowledge, laterality | McCarthy Scales of Children’s Abilities (MSCA) |
| Visual motor processing speed, psycho motor speed, working memory, learning, attention | CogState |
| Fine motor speed and eye-hand coordination | Pegboard |
| Receptive (hearing) vocabulary and verbal ability, | Peabody Picture Vocabulary Test (PPVT) |
| Cognition (Verbal Scale IQ, Performance Scale  IQ, and Full Scale IQ) achievement, adaptive behaviour, executive function, processing speed, working memory, reasoning, verbal comprehension | Wechsler Intelligence Scales for Children (WISC) |
| Cognitive function (verbal comprehension, working memory, visual spatial and processing speed, fluid reasoning, general intellectual functioning) | Wechsler preschool and primary scale for intelligence (WPPSI) IV |
| Psychomotor development, | Kilifi Developmental Inventory |
| Attention (including sustained attention), executive function | Test for everyday attention for children (TEA-Ch). |
| Gross motor, fine motor, visual reception, receptive language, expressive language | Mullen scales of early learning |
| Attention (including sustained attention), cognitive ability, impulsivity, inhibitory control, speed/ response time, focus and vigilance | Tests of Variables of Attention (TOVA) |
| Arithmetic, Spelling, sentence comprehension and word reading | Wide Range Achievement Test (WRAT) |
| Receptive grammar, receptive vocabulary, syntax, lexical semantics, higher level pragmatics | Rivermead behavioural memory test |
| Associative memory | Colour Object Association Test (COAT) |
| Development (gross motor, ﬁne motor, language development, social skills and social development) | Malawi Developmental Assessment Tool (MDAT) |
| Locomotor, personal-social, language, eye and hand coordination, performance, and practical reasoning | Griffith’s Mental Development Scales |
| **Behaviour domain** | |
| Behaviour (Internalising, externalising) and emotional problems | Achenbach Child Behaviour Checklist (CBCL) |
| Socio-emotional problems and Competencies. | Infant and Toddler Socio-Emotional Assessment (ITSEA, or BITSEA –  brief form |
| Socio-Emotional behaviour | Infant and Toddler Socio-Emotional Assessment |
| Socio-Emotional behaviour | Strengths and Difficulties Questionnaire |
| Behavioural manifestations of executive function (Meta-Cognition (Monitor, Organization of Materials, Plan/Organize, Working Memory, Initiate) and Behavioural Regulation (Emotional Control, Shift, Inhibit) | Behaviour Rating Inventory of Executive Function (BRIEF) |
| Adaptive behaviour | Vineland Adaptive Behaviour Scale |
| Adaptive behaviour | Greenspan scale |
| Social emotional behaviour, sensory processing, Adaptive behaviour | Bayley III Social emotional and adaptive behaviour scale |
| Depression, conduct disorder, anxiety, phobia | MINI-KID |
| Emotional, conduct, hyperactivity, peer-problems, ADHD | Strengths and difficulties questionnaire |

**Table 4: Data extraction/data charting tool**

| Author and publication year |
| --- |
| Study title |
| Country |
| Study population |
| Age group and sample size |
| Study design |
| Form of malaria |
| Description of how malaria was assessed |
| Aim of the study |
| Cognitive outcome |
| Cognitive assessment measure |
| Behavioural outcome |
| Behavioural assessment measure |
| Time points of assessment |
| Key findings |
| Most significant findings |
| Conclusions |
| Comments |
|  |

1. The World Bank. How does the World Bank classify countries New York: The World Bank; 2020 [cited 2020 30th April 2020]. Available from: https://datahelpdesk.worldbank.org/knowledgebase/articles/378834-how-does-the-world-bank-classify-countries. [↑](#footnote-ref-1)
