# Supplementary material for: Associations of childhood exposure to malaria with cognition and behavior outcomes: a systematic review protocol
Source: Syst Rev. 2020 Aug 9;9:174. doi: 10.1186/s13643-020-01434-2 (PMC7416398; doi:10.1186/s13643-020-01434-2)
Supplement: Supplementary file 3 — Additional file 3. Supplement [file 13643_2020_1434_MOESM3_ESM.docx]

**Supplement 1**

**Search strategy**

("cognitive"[All Fields] AND (functions [All Fields] OR function [All Fields] OR functioning [All Fields])) OR (neurocognitive[All Fields] OR neurocognitively[All Fields] OR neurocognitives[All Fields] OR neurocognition[All Fields] OR "cognitive"[All Fields] OR cognitives[All Fields] OR cognitively[All Fields] OR "cognition"[MeSH Terms] OR "cognition"[MeSH Terms] OR "cognition"[All Fields] OR "cognition disorders"[MeSH Terms] OR ("cognition disorders"[MeSH Terms] OR ("cognition"[All Fields] AND "disorders"[All Fields])) OR "cognition disorders"[All Fields] OR "child development"[MeSH Terms] OR "child development"[Title/Abstract] OR "child behaviour disorders"[MeSH Terms] OR "child behaviour"[MeSH Terms]) AND ("malaria"[MeSH Terms] OR "malaria"[All Fields])

**Embase**

(('mental disease' OR 'cognition' OR 'behaviour disorder' OR 'developmental disorder' OR 'child development' OR 'neurological disorder' OR 'neurodevelopment' OR 'executive function' OR 'language') AND ('malaria' OR '*falciparum*' OR 'remittent fever') OR 'malaria infection' OR '*plasmodium*') AND ([adolescent]/lim OR [child]/lim OR [infant]/lim OR [newborn]/lim OR [preschool]/lim OR [school]/lim).
